# MiR-1-3p that correlates with left ventricular function of HCM can serve as a potential target and differentiate HCM from DCM

**DOI:** 10.1186/s12967-018-1534-3

**Published:** 2018-06-09

**Authors:** Mengmeng Li, Xiao Chen, Liang Chen, Kai Chen, Jianye Zhou, Jiangping Song

**Affiliations:** State Key Laboratory of Cardiovascular Disease, Fuwai Hospital, National Center for Cardiovascular Diseases, Chinese Academy of Medical Sciences and Peking Union Medical College, 167A Beilishi Road, Xi Cheng District, Beijing, 100037 People’s Republic of China

**Keywords:** HCM, DCM, microRNA, Left ventricular function, Clcn3

## Abstract

**Background:**

MicroRNAs (miRNAs) are non-coding RNAs that function as regulators of gene expression and thereby contribute to the complex disease phenotypes. Hypertrophic cardiomyopathy (HCM) and Dilated cardiomyopathy (DCM) can cause sudden cardiac death and eventually develop into heart failure. However, they have different clinical and pathophysiological phenotype and the expressional spectrum of miRNAs in left ventricles of HCM and DCM has never been compared before.

**Methods:**

This study selected 30 human left ventricular heart samples belonged to three diagnostic groups (Control, HCM, DCM). Each group has ten samples. Based on previous findings, the expression of 13 different microRNAs involving heart failure and hypertrophy (miR-1-3p, miR-10b, miR-21, miR-23a, miR-27a, miR-29a, miR-133a-3p, miR-142-3p, miR-155, miR-199a-3p, miR-199a-5p, miR-214, miR-497) was measured. 17 HCM patients were included as second group to validate the associations.

**Results:**

We found miR-155, miR-10b and miR-23a were highly expressed in both HCM and DCM compared with control. MiR-214 was downregulated and miR-21 was upregulated in DCM but not in HCM. We also identified miR-1-3p and miR-27a expressed significantly different between HCM and DCM and both miRNAs downregulated in HCM. And only miR-1-3p correlated with left ventricular end diastolic diameter (LVEDD) and left ventricular ejection fraction (LVEF) that reflected the cardiac function in HCM. A second HCM group also confirmed this correlation. We then predicted Chloride voltage-gated channel 3 (Clcn3) as a direct target gene of miR-1-3p using bioinformatics tools and confirmed it by Luciferase reporter assay.

**Conclusion:**

Our data demonstrated that different cardiomyopathies had unique miRNA expression pattern. And the expression levels of miR-1-3p and miR-27a had disease-specificity and sensitivity in HCM, whereas only miR-1-3p was significantly associated with left ventricular function in HCM identifying it as a potential target to improve the cardiac function in end-stage HCM. We also provide Clcn3 as a direct target of miR-1-3p which sheds light on the mechanism of HCM.

**Electronic supplementary material:**

The online version of this article (10.1186/s12967-018-1534-3) contains supplementary material, which is available to authorized users.

## Background

Heart failure (HF) is a heart dysfunction disease which associates with high mortality and mobidity [1]. Hypertrophic cardiomyopathy (HCM) and dilated cardiomyopathy (DCM) are two common forms of cardiomyopathy, there is no cure for both these diseases and they gradually cause severe heart failure [2, 3]. HCM is characterized by left ventricular hypertrophy, with predominant involvement of the interventricular septum, affecting 1 in 500 people, whereas DCM is characterized by cardiac ventricular dilation with reduced contractile performance [4, 5]. Genetic investigations have revealed that HCM is caused by mutations in at least ten genes, nine of them encoding for cardiac sarcomeric proteins such as *TNNT2* (cardiac troponin T), *MYH7* (cardiac beta-myosin heavy chain), and *ACTC* (cardiac alpha-actin) which can also cause DCM [6–9]. However, at the end stage of these two diseases, different heart remodeling and molecular changes cause specific clinical and pathological phenotype in HCM and DCM [10–12].

Recent studies have demonstrated miRNAs play a functional role in the progression of heart hypertrophy and heart failure and can influence cell proliferation and modulation both in physiological and pathophysiological ways [13–17]. They are a class of short, non-coding RNAs that regulate the expression of protein coding genes at the post-transcriptional level by binding the 3′ untranslated region of targeted mRNAs [18, 19]. It also has been discovered that miRNAs can serve as potential clinical biomarkers which show specific signature patterns or even work as therapeutic targets for heart disease [20–22]. Since the important role of miRNAs, whether they contribute to the end-stage HCM and DCM progression and reflect the disease state and specificity for these two diseases is still unknown.

We thus aimed to characterize the expression patterns of miRNAs in HCM and DCM, evaluating the expression of 13 miRNAs involved in fibrosis, apoptosis, dilation and hypertrophy (Additional file 1: Table S1) in human left ventricle tissue. We found three miRNAs (miR-155, miR-10b and miR-23a) were increased both in HCM and DCM compared with Control. MiR-214 was downregulated in DCM not HCM whereas miR-21was upregulated in DCM and miR-1-3p and miR-27a were differentially downregulated in HCM compared with DCM. Moreover, we assessed the correlation with cardiac maladaptive remodeling and heart function assessed by ultrasound cardiogram (UCG). Notably, only downregulated miR-1-3p was associated with LVEDD and LVEF in HCM. In order to testify our finding, a second HCM group was included. We found the same correlation trend that the expression of miR-1-3p inversely correlated with LVEDD and directly correlated with LVEF.

Given the important role of miR-1-3p, we next predicted the target genes of miR-1-3p using six algorithms and confirmed Clcn3 as a direct target gene of miR-1-3p which encodes a chloride channel.

Our results demonstrated the different expression pattern of miRNAs which may cause different diseases and the potential of miR-1-3p as a druggable target for the management of hypertrophy.

## Method

### Sample collection

This study selected 30 heart tissue samples belonged to three diagnostic groups (Control, HCM, DCM). All the HCM and DCM samples were from patients underwent heart transplantation because of end-stage heart failure. Control samples were obtained from ten unused donor hearts that were unsuitable for transplantation for past medical history of the donors or for technical reasons. Left ventricles were obtained.

Seventeen HCM patients who underwent heart transplantation were also chosed and collected as a second group from Fuwai Hospital for the validation process.

The cardiac tissue was freshly dissected and frozen in liquid nitrogen. The study protocol was approved by the Human Ethics Committee of Fuwai Hospital of Peking Union Medical College. All the patients were informed and consented for transplantation and clinical investigation.

### RNA extraction and cDNA synthesis

Total RNAs were extracted form heart tissue samples using TRIzol (Invitrogen,USA) according to the manufacture’s protocol. The quality of each RNA sample was assessed by NanoDrop2000 (NanoDrop Technology, USA). All the RNA samples used in the PCR procedures showed a 260/280 nm absorbance ratio between 1.8 and 2.1 and 260/230 ratior exceeds 2.0. Reverse transcription reactions were performed using TransScript miRNA First-Strand cDNA Synthesis SuperMix (TransGen Biotech, China). A PCR System T100 (Bio-Rad, USA) was used to carry out the reverse transcription PCR reactions. The cDNA then was stored in − 80 °C for further experiments.

### miRNA measurement

Quantitative reverse transcription PCR (qRT-PCR) was performed on the 7500 Real-Time PCR System (Applied Biosystems, USA) by using a standard SYBRGreen Real-Time PCR Master Mix (Applied Biosystems, USA) with a 10 μL reaction volume (containing 2 μL cDNA, 0.4 μL miRNA- specific primers,0.4 μL Universal miRNA qPCR primer (TransGen, China), 5 μL of 2 × SYBRGreen Real-Time PCR Master Mix and 2.2 μL of double-distilled water). The reaction mixtures were incubated at 95 °C for 10 min, followed by 40 cycles of 95 °C for 10 s and 60 °C for 1 min. Each reaction had three experimental triplicate and U6 snRNA was used as an internal control for normalization.

### Identification target genes of miR-1-3p

In order to obtain the target genes of miR-1-3p, we used six algorithms including Targetscan, RNA22, PITA, miRDB, miRanda and microRNA.ORG. The same genes predicted by six algorithms were considered as target genes of the miRNA. According to the annotation of the target genes, select the interested gene.

### Cell culture

293T cells were cultured in Dulbecco’s Modified Eagle Medium (DMEM) supplemented with 10% fetal bovine serum and 100 μg/ml penicillin/streptomycin at 37 °C, 5% CO_2_.

### Luciferase reporter assay

Both wild type (WT) and the mutants (MT) of the 3′-UTRs of target were cloned and inserted into the GV272 vector (JiKai, China). The constructs were verified by sequencing. Luciferase reporter assays were performed in 293T cells which were seeded in a 24-well plate one day prior to transfection. Then, both WT and MT plasmids were co-transfected with miR-1-3p plasmids using Lipofectamine 2000 (Invitrogen) respectively. The firefly and Renilla luciferase activities were evaluated simultaneously 24 h after the transfection using the Dual-Luciferase Reporter Assay System (Promega, USA).

### Statistical analysis

Results were expressed as mean values ± SD. The difference between two groups was determined by the non-paired Student t test and one-way analysis of variance (ANOVA) was used to compare the expression differences of miRNAs between control and different patient groups. Receiver operating characteristic curve (ROC) analysis was used to value the area under the curve (AUC) which can evaluate the sensitivity and specificity of miRNAs for specific disease group. AUC > 0.7 was considered acceptable. Differences were considered to be statistically significant when P < 0.05 (two-sided). Pearson’s correlation coefficient and univariable logistic regression analysis were used to assess the correlation between miRNAs and clinical and pathological data of HCM and DCM patients. SPSS 20.0 software, Graphad Prism 5.0 software and MedCalc (version 11) were used for statistical analyses.

## Results

### Patient population

A total of 30 patients (diagnosed as HCM, DCM, Control) were recruited in Fuwai Hospital to get a clear miRNA expression pattern for specific disease. The diagnosis and management of HCM were based on the American Heart Association guidelines and mainly included echocardiographic evidence of maximum left ventricular wall thickness greater than 15 mm in the absence of any other cardiac or systemic disorder producing a comparable grade of hypertrophy [23]. And patients with DCM were established when chambers were dilated combining with a systolic LVEF ≤ 35% [24]. Patients characteristics were summarized in Table 1.

**Table 1:** Clinical data of HCM and DCM patients

	HCM	DCM	p
Sample number	10	10	
Mean age (years)	39 ± 14	47 ± 14	0.23
Male sex (%)	80%	80%	1
BMI (kg/m^2^)	22 ± 4.31	20 ± 1.51	0.26
UCG			
LVEF (%)	30.29 ± 10.42	25 ± 6.37	0.21
LVEDD (mm)	61.6 ± 14.04	68.6 ± 7.98	0.21
IVS (mm)	13.98 ± 3.16	8.9 ± 1.36	0.001**
LVPW (mm)	10.48 ± 3.33	8.6 ± 1.28	0.141
Comorbidities			
Hypertension	20% (2)	0	0.151
Ventricular tachycardia	40% (4)	40% (4)	1
Diabetes	10% (1)	50% (5)	0.054
Myocardial infarction	0	0	1
Atrial fibrillation	40% (4)	50% (5)	0.673
Medications			
Digoxin	50% (5)	70% (7)	0.388
β-blocker	80% (8)	40% (4)	0.074
Aldosterone antagonist	70% (7)	50% (5)	0.388
ACE inhibitor/ARB	20% (2)	20% (2)	1
Mean heart weight (g)	542.1 ± 144.88	380.9 ± 61.01	0.01*

Data are expressed as mean ± SD

*BMI* body mass index; *UCG* ultrasound cardiogram; *LVEF* left ventricular ejection fraction; *LVEDD* left ventricular end diastolic diameter; *IVS* interventricular septum; *LVPW* left ventricular posterior wall; *ARB* Angiotension receptor blocker

*p < 0.05, **p < 0.01, ***p < 0.001

Seventeen HCM patients were also recruited in Fuwai Hospital to validate our finding. Patients characteristics were summarized in Additional file 1: Table S2.

### MiR-155, miR-10b, miR-23a were upregulated in HCM and DCM

**Fig. 1:**
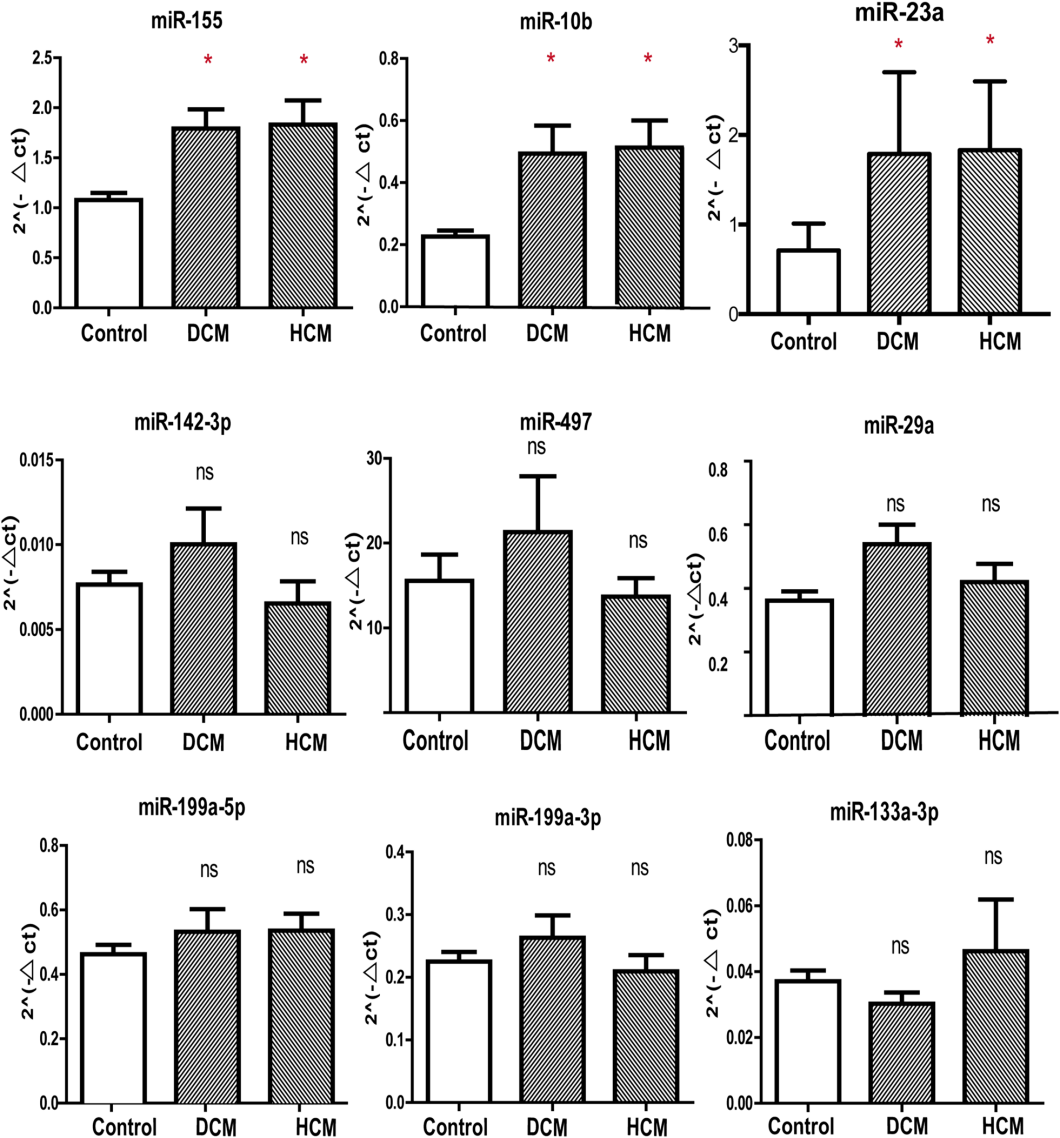
Expression of miRNAs in HCM, DCM and Control LV. The sample number of each group is 10 and p values were obtained with ANOVA test between three groups. *p < 0.05, **p < 0.01, ***p < 0.001, *ns* not significant (p ≥ 0.05). *HCM* hypertrophic cardiomyopathy; *DCM* dilated cardiomyopathy

MiRNA levels were assessed in HCM, DCM, Control groups and each including 10 left ventricular tissue samples. Among the 13 miRNAs analyzed, miR-155, miR-10b and miR-23a were found significantly increased in HCM and DCM samples compared with Control (Fig. 1). These three miRNAs showed the similar expression pattern in HCM and DCM samples. No significant differences were observed for the expression of miR-142-3p, miR-497, miR-29a, miR-199a-5p, miR-199a-3p and miR-133a-3p in HCM, DCM compared with Control groups (Fig. 1).

### The expression of miR-214, 21, 27a, 1-3p showed disease specificity

**Fig. 2:**
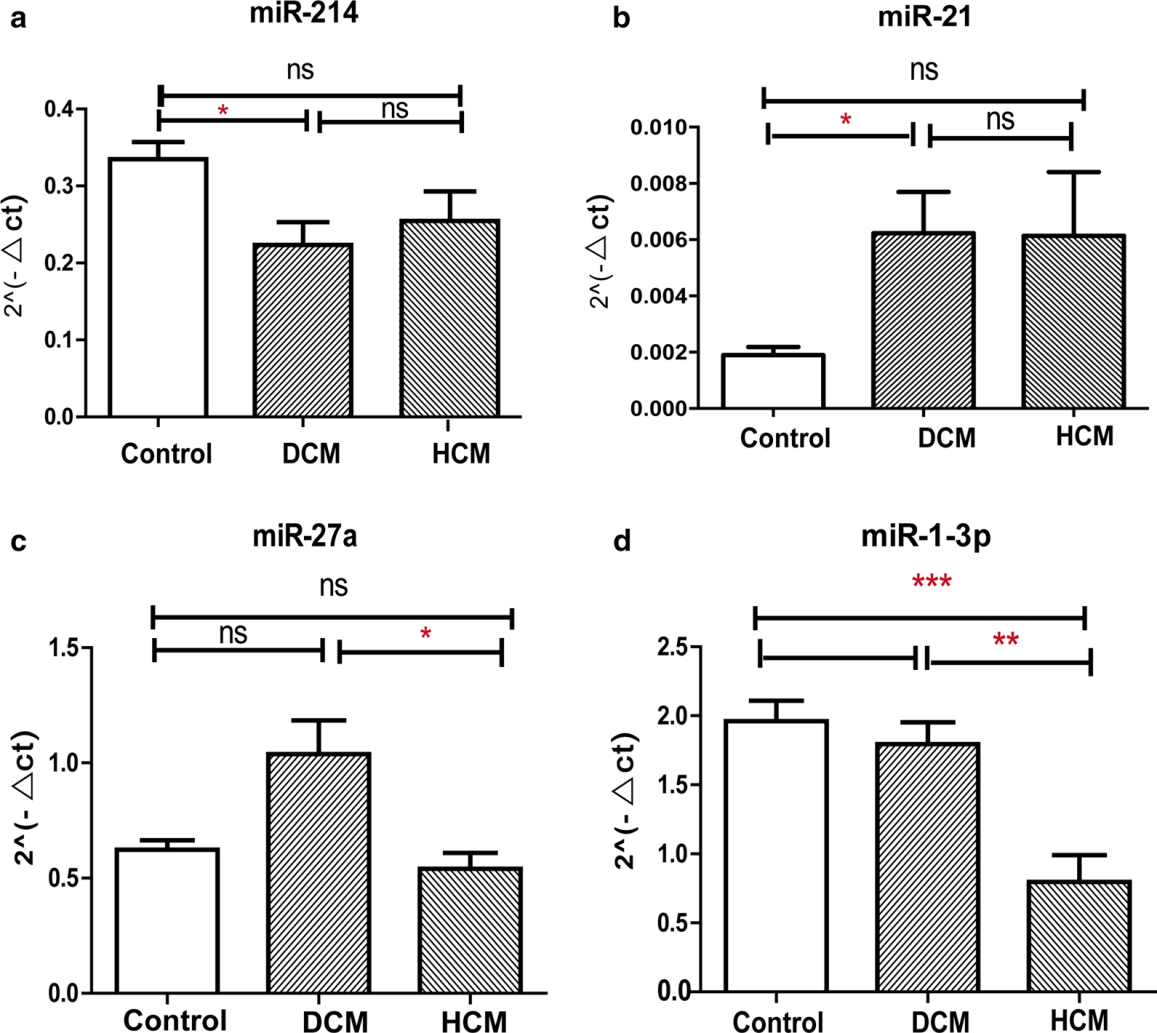
Different expression pattern of miRNAs for HCM and DCM. **a** miR-214 was detected downregulated in DCM compared with Control. **b** miR-21 was upregulated in DCM compared with Control. **c**, **d** miR-27a and miR-1-3p were upregulated in HCM compared with DCM. The sample number of each group is 10 and p values were obtained with ANOVA test *p < 0.05, **p < 0.01, ***p < 0.001, *ns* not significant (p ≥ 0.05). *HCM* hypertrophic cardiomyopathy; *DCM* dilated cardiomyopathy

Our study also found some miRNAs expressed differently in HCM and DCM. MiR-214 was only downregulated in DCM group and the expression of miR-21 significantly upregulated in DCM (Fig. 2a, b). The expression levels of miR-214 and miR-21 between HCM and Control showed no significance. Two miRNAs expressed significantly different between HCM and DCM. The expression level of miR-27a in DCM increased compared with HCM group. MiR-1-3p was found significantly downregulated in HCM compared with DCM and Control group with p value being 0.003 and 0.001 respectively (Fig. 2c, d).

### Receiver operating characteristic (ROC) curves analysis

**Fig. 3:**
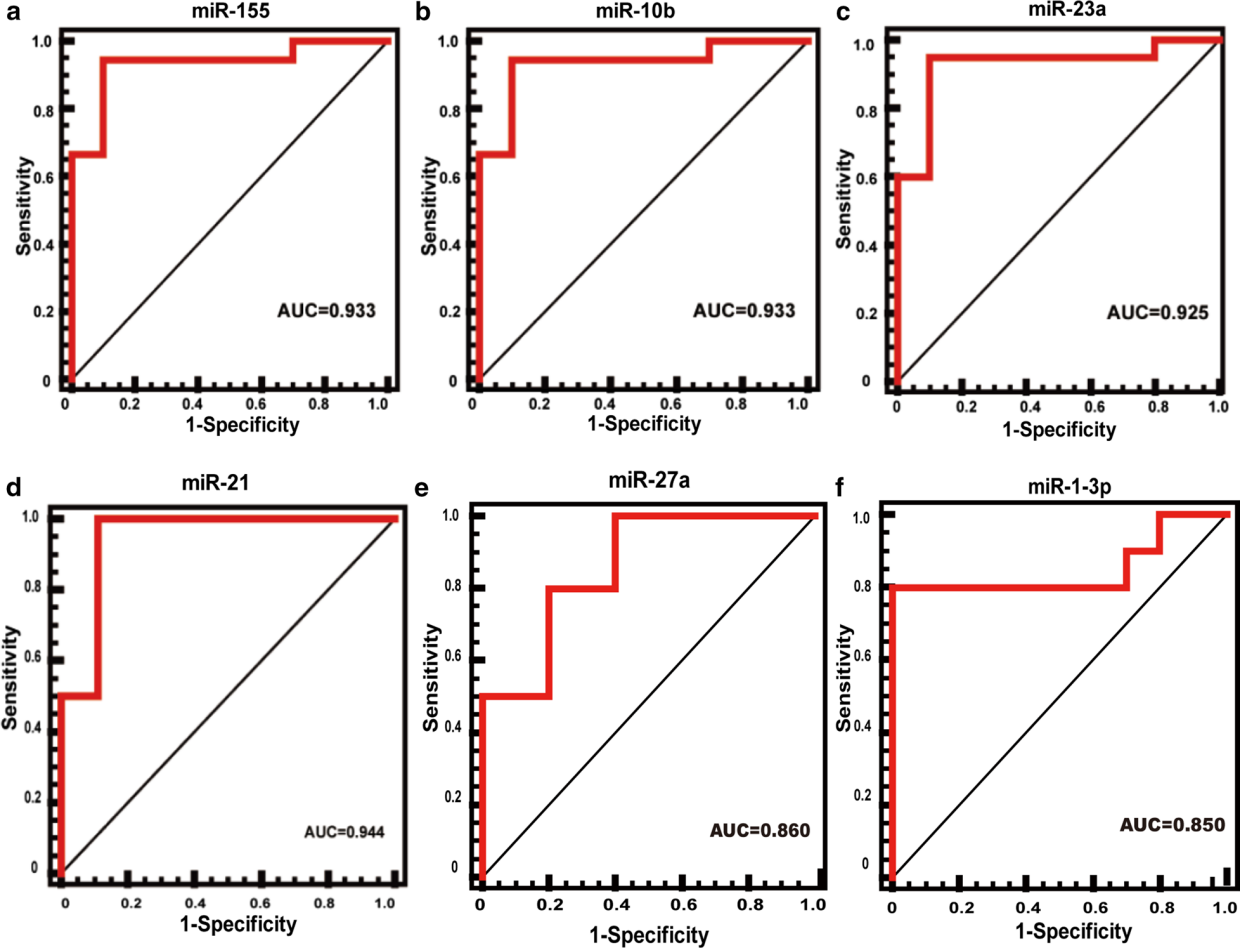
ROC curve analysis of microRNAs. **a**–**c** ROC curve analysis of miR-155, miR-10b and miR-23a showed high sensitivity and specificity in discriminating disease group (HCM, DCM) with AUC ranged from 0.925 to 0.933. **d** ROC curve analysis of miR-21 showed AUC was notably reaching 0.944 which could distinguish DCM from Control. **e**–**f** ROC curve analysis of miR-27a and miR-1-3p showed high sensitivity and specificity in discriminating HCM from Control. Area under the curve (AUC) > 0.7 was considered accepted. *HCM* hypertrophic cardiomyopathy; *DCM* dilated cardiomyopathy

We were able to find that the expression of miR-10b, miR-23a and miR-155 increased significantly in disease groups (HCM, DCM). To test whether these three miRNAs could distinguish disease groups from Control, ROC curve analysis was performed. The specificity and negative predictive value for three miRNAs were all greater than 0.9 allowing for the discrimination between diseases and Control group (Fig. 3a–c). ROC analysis showed that AUC was smaller than 0.7 for miR-214 and AUC was notably reaching 0.944 for miR-21 which could distinguish DCM from Control (Fig. 3d). MiR-27a and miR-1-3p was downregulated in HCM compared with DCM. The ACU was 0.860 and 0.850 respectively (Fig. 3e, f).

### Association of miRNA level with left heart function

After the expression differences of miRNAs in HCM and DCM had been determined, we then evaluated whether any changed miRNA was correlated with baseline clinical and pathological data of HCM and DCM (Tables 2, 3). Both HCM and DCM predominately affect left ventricle and the left heart function of both diseases is very serious. LVEF and LVEDD abstained by UCG are common prognostic data for left heart function. The level of miR-1-3p expression inversely correlated with LVEDD (r = − 0.773; p = 0.009) and directly correlated with LVEF(r = 0.737; p = 0.015) in HCM.

**Table 2:** Pearson correlations between changed miRNAs and clinical and pathological data of HCM

	LVEDD		LVEF		IVS		LVPW		HW	
	r Value	p Value	r Value	p Value	r Value	p Value	r Value	p Value	r Value	p Value
miR-155	− 0.305	0.391	0.219	0.543	− 0.605	0.064	− 0.537	0.110	− 0.212	0.556
miR-10b	− 0.020	0.957	− 0.054	0.883	0.120	0.740	− 0.445	0.197	0.226	0.531
miR-23a	− 0.230	0.522	0.036	0.921	0.017	0.964	− 0.467	0.174	− 0.058	0.874
miR-27a	− 0.588	0.074	0.440	0.203	0.325	0.360	− 0.049	0.893	− 0.134	0.712
miR-1-3p	− 0.773	0.009**	0.737	0.015*	0.249	0.488	− 0.217	0.546	− 0.536	0.110

Pearson correlation coefficient (r) and relative computed p value were assessed

*p < 0.05, **p < 0.01, ***p < 0.001. Abbreviations were listed in Table 1

**Table 3:** Pearson correlations between changed miRNAs and clinical and pathological data of DCM

	LVEDD		LVEF		IVS		LVPW		HW	
	r Value	p Value	r Value	p Value	r Value	p Value	r Value	p Value	r Value	p Value
miR-155	− 0.488	0.153	0.485	0.155	0.587	0.075	0.413	0.236	0.597	0.068
miR-10b	0.323	0.363	− 0.415	0.232	0.007	0.985	0.014	0.969	− 0.166	0.647
miR-23a	0.122	0.754	− 0.026	0.947	− 0.500	0.171	− 0.293	0.444	− 0.376	0.319
miR-214	0.294	0.409	− 0.394	0.260	0.125	0.731	0.188	0.603	0.110	0.762
MiR-21	− 0.590	0.872	0.157	0.665	− 0.116	0.749	− 0.032	0.929	− 0.169	0.641

Pearson correlation coefficient (r) and relative computed p value were assessed

*p < 0.05, ** p < 0.01, ***p < 0.001. Abbreviations were listed in Table 1

However, the univariable logistics analysis showed miR-1-3p was not associated with age and commordities including hypertension, ventricular tachycardia, diabetes, myocardial infarction and atrial fibrillation. In addition, no significant results were found between miRNAs with heart weight.

**Fig. 4:**
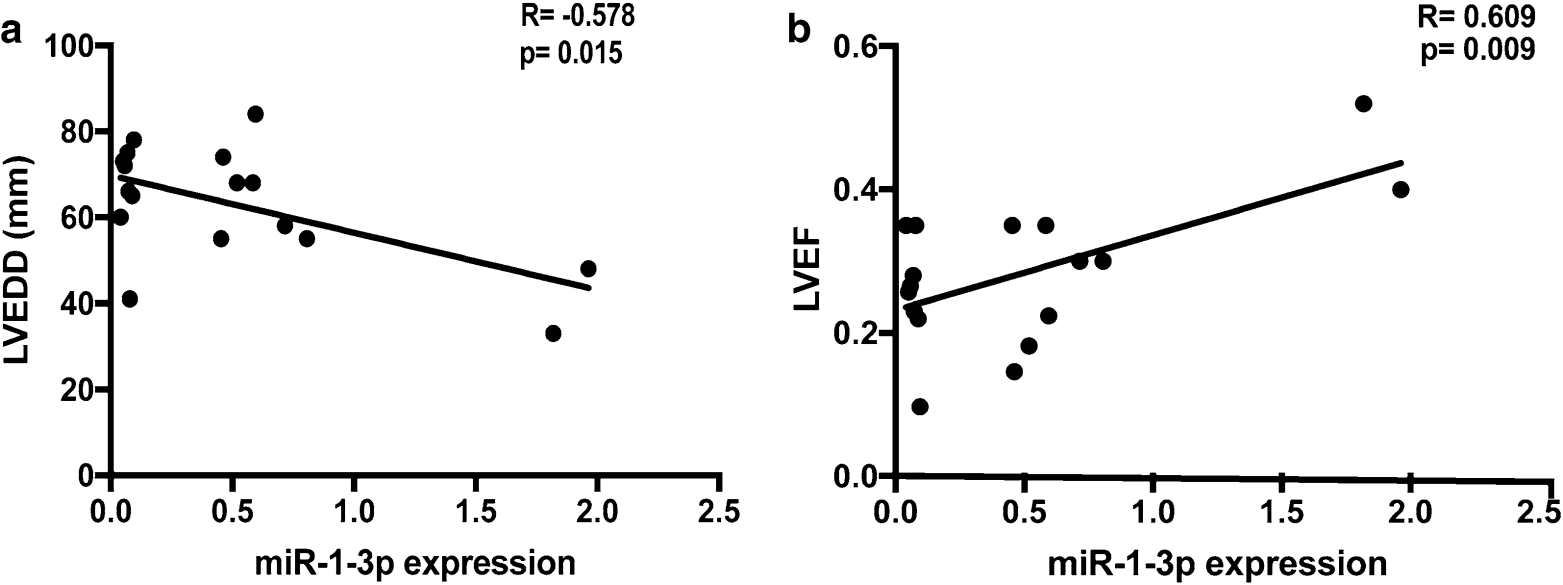
Validate the association between miR-1-3p and indices of left heart function in a second HCM group. **a**, **b** The expression value of miR-1-3p in 17 HCM patients inversely correlated with LVEDD and directly correlated with LVEF. The Pearson correlation coefficient (r) and the relative computed p value are displayed on the graphs

In order to testify our finding, we used a second HCM group to validate the correlation between the expression of miR-1-3p and LVEF and LVEDD. We found the same correlation trend that the expression of miR-1-3p inversely correlated with LVEDD (r = 0.015; p = − 0.578) and directly correlated with LVEF (r = 0.009; p = 0.609) (Fig. 4).

### Prediction targets of miR-1-3p

Since the function of miRNAs is repressing target genes expression, we use 6 algorithms including Targetscan, RNA22, PITA, miRDB, miRanda and microRNA.ORG to predict the targets of miR-1-3p. Given that a gene predicted by multiple algorithms is more likely to be the target of miR-1-3p. 7 target genes were predicted by all 6 algorithms (Additional file 1: Table S3). According to the annotation of genes, we found Clcn3 (Chloride voltage-gated channel 3) as a promising target of miR-1-3p.

### Validation of Clcn3 as a direct target of miR-1-3p

To establish whether the interaction of Clcn3 and miR-1-3p is direct, we applied Luciferase reporter assay. We cloned the 3′UTR of Clcn3 into GV272 vector after firefly luciferase coding region. The relative luciferase activity in the Clcn3.

**Fig. 5:**
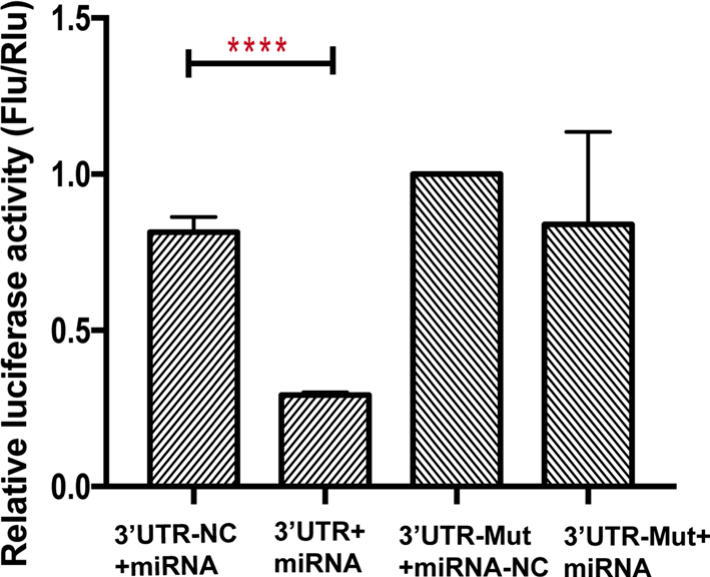
Validation of Clcn3 as a direct target of miR-1-3p. Luciferase reporter assay in 293T cells. 3′UTR sequences form Clcn3 containing predicted miR-1-3p recognition sequences were cloned downstream of luciferase. The results demonstrated as firefly luciferase activity versus Renilla luciferase activity. The relative activity was reduced by a co-transfected miR-1-3p plasmid. Student’s *t* test was used to analyze the results, n = 3 independent experiments. *p < 0.05, **p < 0.01, ***p < 0.001, ****p < 0.0001

3′UTR+miRNA was significantly lower than that in the Clcn3 3′UTR-NC+miRNA group, while Clcn3 3′UTR-Mut+miRNA-NC group had a similar luciferase activity compared with Clcn3 3′UTR-Mut+miRNA (Fig. 5).These results indicated that miR-1-3p can repress firefly luciferase activity by binding Clcn3 3′ UTR which mean that miR-1-3p directly targets Clcn3.

## Discussion

MiRNAs are important regulators of gene expression and cellular processes. Recently, multiple researches have reported miRNAs express differently in human heart disease and these changes may contribute to heart disease pathogenesis and phenotype [25]. The obvious importance of miRNAs predicates them to be potential therapy target for congenital and acquired heart disease [19].

In this study we have assessed 13 miRNAs mainly involved in cardiac hypertrophy and heart failure to identify the specific miRNA expression pattern for end-stage HCM and DCM using left ventricular tissue form patients underwent heart transplantation.

Among 13 miRNAs, we identified that 3 miRNAs (miR-155, miR-10b and miR-23a) were significantly increased in both HCM and DCM, and these three upregulated miRNAs all had an ROC curve that distinguished the disease group (HCM and DCM) from the Control (AUC ranges from 0.925 to 0.933). As the samples we used in this study were all from end-stage heart failure patients, so the same changed miRNAs in HCM and DCM may reflect the heart failure similarity. The results were consistent with previous reports [26–28]. Martin et al. have reported angiotensin II type 1 receptor (AT1) as a target of miR-155 [29]. MiR-10b targeted the 3′-untranslated region of TBX5 to repress its expression [30] and the muscle specific ring finger protein1 was identified to be a target of miR-23a [28]. These common regulated miRNAs may be the therapeutic target for the treatment of heart failure in the near future.

However, the expression of miR-142-3p, miR-497, miR-29a, miR-199a-5p, miR-199a-3p and miR-133a-3p showed no significance in our patients. Not all the unchanged miRNAs are in agreement with other studies. These findings may reflect the differences in myocardial regions sampled and patients selected.

Furthermore, the expression of miR-214, miR-21, miR-27a and miR-1-3p showed disease specificity, which may provide new avenue for differentiating the two conditions. The expression of miR-214 was downregulated in DCM whereas miR-21 was upregulated in DCM compared with Control and no significance difference was found between HCM and Control. The ROC curve of miR-21 also showed a high sensitivity and specificity with an AUC of 0.944. The role of miR-21 in myocardial fibrosis has been widely studied. It has been reported that miR-21 is upregulated in cardiac fibroblasts in heart failure [31]. In a mouse model, it has been demonstrated that silencing of miR-21 could regulate fibrosis and reduce heart dysfunction in vivo [32]. The high expression of miR-21 in DCM is accordance with the abundant myocardial fibrosis found in DCM by cardiovascular magnetic resonance (CMR) and pathology. And the presence of myocardial fibrosis may be one of the important factor for the failure to respond to treatment for end-stage DCM patients [33].

Notably, we found miR-27a and miR-1-3p expressed differently between HCM and DCM group. The ROC curve of both miRNAs demonstrated high specificity and sensitivity. The main function of miR-27a is to regulate endothelial cell repulsion and vessel formation and it is highly expressed in endothelial cells [34]. Here, our result showed the expression of miR-27a was variable in HCM and DCM but no significance compared with Control. The downregulation of miR-1-3p was significant compared with DCM and Control and it may work as a negative regulator of cardiac hypertrophy. And the expression of miR-1-3p between DCM and Control showed no apparent change which predicts miR-1-3p may regulate gene expression changes that eventually cause different pathological and clinical phenotypes. Our study is accordance with the miRNA profiling conducted on the mouse of end-stage hypertrophy [27].

The major finding from this study is that the expression of miR-1-3p correlated negatively with LVEDD and positively with LVEF in HCM. LVEDD and LVEF assessed by UCG are direct clinical component of left ventricular function. In the HCM patients, the downregulation of miR-1-3p is associated with the larger LVEDD and the smaller LVEF which reflect the severe heart function. In order to validate the correlation, we also use 17 HCM LV samples to testify our finding. We found the same correlation trend. The results indicate that miR-1-3p may work as a potential target in end-stage HCM patients to relieve heart function.

Based on these intriguing findings, we further predicted the target genes of miR-1-3p by using 6 algorithms. Clcn3 was predicted as a promising target gene. The interaction was testified by Luciferase reporter assay which demonstrated that Clcn3 is the direct target of miR-1-3p. Many studies have demonstrated Clcn3 plays an important role in cardiac and vascular remodeling during myocardial hypertrophy [35, 36]. Heart- specific inducible Clcn3 knock-out mice produced myocardial hypertrophy and significantly reduced cardiac function (marked shortening of LVEF and significant increasing in LV chamber cavity) compared with control mice [37].

All of these results taken together demonstrated the important role of miR-1-3p in HCM.

## Conclusion

We demonstrated the different expression patterns of miRNAs between HCM and DCM which suggest that miRNA may play an important role in pathological-specific gene expression alterations. MiR-1-3p correlated with left ventricular function in HCM patients identifying it as a potential therapy target for cardiac dysfunction. We also confimed Clcn3 as a direct target of miR-1-3p which may provide novel therapeutic tools for the treatment of myocardial hypertrophy.

## Additional file

**Additional file 1**

## Abbreviations

miRNAs: microRNAs
HCM: hypertrophic cardiomyopathy
DCM: dilated cardiomyopathy
LVEDD: left ventricular end diastolic diameter
LVEF: left ventricular ejection fraction
Clcn3: chloride voltage-gated channel 3
HF: heart failure
UCG: ultrasound cardiogram
