# Supplementary material for: MiR-1-3p that correlates with left ventricular function of HCM can serve as a potential target and differentiate HCM from DCM
Source: J Transl Med. 2018 Jun 9;16:161. doi: 10.1186/s12967-018-1534-3 (PMC5994246; doi:10.1186/s12967-018-1534-3)
Supplement: Supplementary file 1 — Additional file 1 [file 12967_2018_1534_MOESM1_ESM.docx]

**Supplementary data**

**Table S1 The primer sequences and function of miRNAs**

| microRNA | Primer sequence | Function |
| --- | --- | --- |
| miR-1-3p | 5′-TGGAATGTAAAGAAGTATGTAT-3′ | Muscle specific miRNAs; Involve in induction of hypertrophy |
| miR-10b | 5′-TACCCTGTAGAACCGAATTTGTG-3′ | Regulates mature cardiomyocyte function |
| miR-21 | 5′-TAGCTTATCAGACTGATGTTGA-3′ | Regulates cell growth |
| miR-23a | 5′-ATCACATTGCCAGGGATTTCC-3′ | Induces hypertrophic response in cardiomyocytes |
| miR-27a | 5′-TAGCACCATCTGAAATCGGTTA-3′ | Regulates endothelial cell repulsion and vessel formation |
| miR-29a | 5′-TAGCACCATCTGAAATCGGTTA-3′ | Contributes to scar formation and fibrosis |
| miR-133a-3p | 5′-TTTGGTCCCCTTCAACCAGCTG-3′ | Plays an important role in cardiac development |
| miR-142-3p | 5′-TGCGGTGTAGTGTTTCCTACTT-3′ | Modulates cardiac formation and function |
| miR-155 | 5′-TTAATGCTAATCGTGATAGGGGT-3′ | Suppresses the level of Ang II type 1 in fibroblasts |
| miR-199a-3p | 5′-ACAGTAGTCTGCACATTGGTTA-3′ | Regulates cell proliferation and survival |
| miR-199a-5p | 5′-CCCAGTGTTCAGACTACCTGTTC-3′ | Associates with hypertrophy in cardiomyocytes |
| miR-214 | 5′-ACAGCAGGCACAGACAGGCAGT-3′ | Protects heart from ischemic injury |
| miR-497 | 5′-GCAGCACACTGTGGTTTGTAAAA-3′ | Plays an important role in the development of heart failure |

**Table S2: Clinical characteristics of patients in a second HCM group**

|  | HCM |
| --- | --- |
| Sample number | 17 |
| Mean age (yrs) | 38±14 |
| Male sex, % | 64.7% |
| UGG |  |
| LVEF (%) | 28.39±9.79 |
| LVEDD (mm) | 63.12±13.16 |
| IVS (mm) | 11.39±4.61 |
| LVPW (mm) | 11.25±4.44 |
| Comorbidities |  |
| Hypertension | 11.76%（2） |
| Ventricular tachycardia | 35.29%（6） |
| Diabetes | 11.76%（2） |
| Myocardial infarction | 0 |
| Atrial fibrillation | 35.29%（6） |
| Medications |  |
| Digoxin | 52.94%（9） |
| β-blocker | 82.35%（14） |
| Aldosterone antagonist | 70.59%（12） |
| ACE inhibitor／ARB | 35.29%（6） |
| Mean heart weight (g) | 526.76±173.99 |

Data are expressed as mean ± SD.

BMI= body mass index; UGG= ultrasound cardiogram; LVEF=left ventricular ejection fraction; LVEDD = left ventricular end diastolic diameter; IVS= interventricular septum; LVPW= left ventricular posterior wall; ARB= Angiotension receptor blocker

* = p < 0.05, ** = p <0.01, *** = p < 0.001

**Table S3: Summary of 7 genes predicted by all 6 algorithms as targets of miR-1-3p.**

| Gene name | Full name | Function annotation |
| --- | --- | --- |
| DDX5 | DEAD-box helicase 5 | Involved in the DNA replication and is required for cancer cell proliferation |
| NDRG3 | NDRG family member 3 | Tumor-related gene |
| CLCN3 | chloride voltage-gated channel 3 | Plays an important role in cardiac and vascular remodeling during myocardial hypertrophy |
| RASA1 | RAS p21 protein activator 1 | Regulates cellular proliferation and differentiation |
| HS3ST3B1 | heparan sulfate-glucosamine 3-sulfotransferase 3B1 | Mediates the process of epithelial-mesenchymal transition |
| SPRED1 | sprouty related EVH1 domain containing 1 | Regulates activation of the MAP kinase cascade |
| JARID2 | jumonji and AT-rich interaction domain containing 2 | A cell cycle regulator |
